# Efficacy and Safety Study of Wearable Cyborg HAL (Hybrid Assistive Limb) in Hemiplegic Patients With Acute Stroke (EARLY GAIT Study): Protocols for a Randomized Controlled Trial

**DOI:** 10.3389/fnins.2021.666562

**Published:** 2021-07-02

**Authors:** Hiroki Watanabe, Aiki Marushima, Hideki Kadone, Yukiyo Shimizu, Shigeki Kubota, Tenyu Hino, Masayuki Sato, Yoshiro Ito, Mikito Hayakawa, Hideo Tsurushima, Kazushi Maruo, Yasushi Hada, Eiichi Ishikawa, Yuji Matsumaru

**Affiliations:** ^1^Department of Neurosurgery, Faculty of Medicine, University of Tsukuba, Tsukuba, Japan; ^2^Center for Cybernics Research, University of Tsukuba, Tsukuba, Japan; ^3^Department of Rehabilitation Medicine, Faculty of Medicine, University of Tsukuba, Tsukuba, Japan; ^4^Department of Orthopaedic Surgery, Faculty of Medicine, University of Tsukuba, Tsukuba, Japan; ^5^Division of Stroke Prevention and Treatment, Faculty of Medicine, University of Tsukuba, Tsukuba, Japan; ^6^Department of Biostatistics, Faculty of Medicine, University of Tsukuba, Tsukuba, Japan

**Keywords:** wearable cyborg, Hybrid Assistive Limb, acute stroke, independent walking, functional ambulation category, randomized controlled trial, gait training

## Abstract

We hypothesized that gait treatment with a wearable cyborg Hybrid Assistive Limb (HAL) would improve the walking ability of patients with hemiparesis after stroke. This study aims to evaluate the efficacy and safety of gait treatment using HAL versus conventional gait training (CGT) in hemiplegic patients with acute stroke and establish a protocol for doctor-initiated clinical trials for acute stroke. We will enroll patients with acute stroke at the University of Tsukuba Hospital. This study is a single-center, randomized, parallel-group, controlled trial (HAL group, *n* = 20; control group, *n* = 20) that will include three phases: (1) pre-observation phase (patient enrollment, baseline assessment, and randomization); (2) treatment phase (nine sessions, twice or thrice per week over 3−4 weeks; the HAL and control groups will perform gait treatment using HAL or CGT, respectively, and finally (3) post-treatment evaluation phase. The Functional Ambulation Category score will be the primary outcome measure, and the following secondary outcome measures will be assessed: Mini-Mental State Examination, Brunnstrom recovery stage of lower limbs, Fugl–Meyer assessment of lower limbs, 6-min walking distance, comfortable gait speed, step length, cadence, Barthel Index, Functional Independence Measure, gait posture, motion analysis (muscle activity), amount of activity (evaluated using an activity meter), stroke-specific QOL, and modified Rankin Scale score. The baseline assessment, post-treatment evaluation, and follow-up assessment will evaluate the overall outcome measures; for other evaluations, physical function evaluation centered on walking will be performed exclusively, excluding ADL and QOL scores. This study is a randomized controlled trial that aims to clarify the efficacy and safety of gait treatment using HAL compared with CGT in hemiplegic patients with acute stroke. In addition, we aim to establish a protocol for doctor-initiated clinical trials for acute stroke based on the study results. If our results demonstrate the effectiveness of the proposed treatment regarding outcomes of patients with hemiplegic acute stroke, this study will promote the treatment of these patients using the HAL system as an effective tool in future stroke rehabilitation programs. The study protocol was registered with the Japan Registry of Clinical Trials on October 14, 2020 (jRCTs032200151).

## Introduction

The Global Burden of Disease (GBD) study (2016) reported that the lifetime risk of stroke onset from 25 years old was approximately 25%; thereafter, many people might suffer from stroke during their lifetime (GBD 2016 Lifetime Risk of Stroke Collaborators, 2018). Stroke is a major cause of death and disability globally (Rigby et al., 2009; Campbell and Khatri, 2020). In Japan, stroke is the fourth leading cause of death and the second leading cause of social-care needs (Ministry of Health Labour and Welfare, 2019a, b). Patients with stroke often exhibit cognitive and higher brain dysfunction, in addition to movement disorders, sensory disorders, and gait disorders, which represent a serious social problem because of the deterioration of activities of daily living (ADL) and quality of life (QOL) (Langhorne and Kwakkel, 2011; Fryer et al., 2016; Mehrholz et al., 2018, 2020).

Restoration of gait function is reported to occur primarily within the first 11 weeks after stroke (Jørgensen et al., 1995a), and intensive gait treatment should begin in the acute phase, which is within 4 weeks after stroke onset (Jørgensen et al., 1995b). Early treatment, particularly task-specific training programs for the upper and lower limbs, is important in stroke rehabilitation (Duncan et al., 2011; Waddell et al., 2017). Several previous studies have shown that intensive, repetitive, task-specific training initiated early after stroke onset accelerates functional recovery and improves the final motor outcomes, including gait function (Kwakkel et al., 1999, 2004; Langhorne et al., 2009; Langhorne and Kwakkel, 2011). Moreover, the recovery of walking ability and independent walking are common therapeutic goals after a major stroke (Dobkin, 2005; Fulk et al., 2017). Since the 1990s, robot-assisted gait training (RAGT) has been introduced clinically and has been used in stroke rehabilitation (Hesse et al., 2003). A systematic review of electromechanical-assisted training and RAGT reported in 2020 found that the combination of these two modalities with physical therapy contributed to the improvement of independent walking after stroke (Mehrholz et al., 2020). It has been suggested that it may be most effective for patients who cannot walk within 3 months of stroke onset (Mehrholz et al., 2020).

The wearable cyborg Hybrid Assistive Limb (HAL) is the world’s first cyborg-type wearable device for supporting, improving, and expanding the wearer’s physical functions based on the detection of bioelectrical signals (BES) on the skin surface while a wearer attempts to generate muscle force (Lee and Sankai, 2005; Suzuki et al., 2007). BES sensor is a system to detect the activities of the extensor and the flexor muscle of the knee and the hip joint. In addition to BES sensor, HAL is equipped with a sensing system of angular sensors on the knee and hip joint to measure the joint angles, and a floor reaction force sensors to measure reaction force from the heel and toe area of foot. The power unit of HAL generates and controls assistive joint torques of the knee and hip to support wearer’s gait movement based on the these sensor of BES, joint sensor and foot reaction force sensors (Kawamoto et al., 2009; Sankai and Sakurai, 2018). Treatment using HAL is based on the principles of the interactive biofeedback hypothesis (Sankai and Sakurai, 2018). It uses a hybrid control system composed of two cybernic control modes (voluntary and autonomous) (Kawamoto et al., 2009). The voluntary control mode is activated by the wearer’s BES and provides physical support and action through voluntary intention (Kawamoto and Sankai, 2005). However, patients with stroke-induced hemiparesis cannot move their extremities. In this setting, HAL can support movement by utilizing the very weak BES detected on the skin surface for autonomous activation. This movement and exercise may enhance the recovery of the impaired neuronal network, and interactive biofeedback may further promote the appropriate reorganization of the neuronal network (Morishita and Inoue, 2016; Sankai and Sakurai, 2018).

The single-leg version of the HAL is a new wearable cyborg designed to be used by patients with hemiplegia (Kawamoto et al., 2009; Watanabe et al., 2014, 2017). In addition, many clinical studies have been conducted on the application of HAL to both legs in patients with stroke (Sczesny-Kaiser et al., 2019; Yokota et al., 2019; Taki et al., 2020). In Japan, a doctor-initiated clinical trial of the single-leg version of HAL as a medical device application (HAL-TS01) in patients with stroke-induced hemiplegia in the late recovery phase is currently underway (Tsurushima et al., 2019). To the best of our knowledge, one randomized controlled trial (RCT) has compared HAL with conventional therapies regarding patient outcomes after subacute stroke (within 5 weeks after stroke onset) (Wall et al., 2020). In their RCT, Wall et al. (2020) reported no between-group difference in any outcome, including the functional ambulation category (FAC) score after intervention or at 6 months after stroke, despite the extra resources required for the HAL training. In addition, younger age was determined as the only factor that enhances independent walking. However, the authors indicated that there was room for consideration of HAL treatment methods (treadmill or overground gait training) based on indications and other factors.

The results of our previous non-randomized clinical trial suggested that the HAL treatment group showed a significant improvement in FAC after nine sessions compared with the conventional treatment group (Watanabe et al., 2020). Furthermore, examination of changes in FAC on a weekly basis revealed a significant group × time point interaction in FAC of the HAL treatment group, suggesting that independent walking may be improved at an early stage [unpublished data]. However, because the study did not have an RCT design, it was thought that there were many biases and variations, as well as a problem in ensuring comparability. Therefore, we planned an RCT to compare the efficacy and safety of HAL treatment with those of conventional gait training (CGT) in patients with hemiplegic acute stroke.

## Methods and Analysis

### Objective

The purpose of this RCT is to evaluate the efficacy and safety of gait treatment using HAL versus CGT among hemiplegic patients with acute stroke and collect outcome data such as improvement of independent walking, gait speed, and endurance.

### Design and Setting

This is a single-center, randomized, parallel-group, controlled trial. Participants will be randomly assigned to either the HAL or CGT group and undergo gait training (with or without HAL) for 20 min per day, twice or thrice per week, over 3−4 weeks (i.e., nine sessions during the study period).

### Participants

Patients admitted to the University of Tsukuba Hospital with acute-onset stroke after October 2020 will be invited to participate in this study.

### Inclusion Criteria

Patients who meet all of the following inclusion criteria will be eligible for enrollment in this study.

1) Patients who can provide consent to participate and sign the form by themselves. However, if writing is difficult, a writer will be provided.
2) Patients aged ≥16 years. If the patient is younger than 20 years, the signature of a parent or guardian is also needed, in addition to the signature of the person him/herself.
3) Patients at a stage within 7−21 days after stroke onset.
4) Patients with an FAC score of 0–2.
5) Patients who can undergo gait training using a mobile suspension system (All-In-One Walking Trainer, Ropox A/S, Denmark).
6) Patients who can be hospitalized for ≥4 weeks from the start of the study.
7) Patients with an FAC score of ≥4 before stroke onset.
8) The HAL group will comprise patients who can wear the lower-limb version of HAL. (Height is assumed to be 150−190 cm; however, the limitation to the use of HAL is not height but fit (body-size parameters such as thigh length, lower leg length, waist width, etc.).

### Exclusion Criteria

Patients who meet any of the following criteria will be excluded from the study.

1) Patients who have difficulty performing voluntary limb movements according to instructions because of consciousness disorder or cognitive dysfunction.
2) Patients who have difficulty in performing joint exercises or wearing HAL because of complications such as heart disease and musculoskeletal system dysfunction, which are problematic during exercise.
3) Patients in whom the bio-electrode of the HAL system cannot be attached because of skin diseases.
4) Patients who participated in other studies within 12 weeks of the start of this study.
5) Patients who are judged to be medically unstable by the principal investigator or member doctors after comprehensively considering physical findings, blood test findings, etc.

### Allocation and Blinding

Enrolled patients will be randomly assigned to either the HAL group or the CGT (control) group at a 1:1 allocation ratio via the minimization method using the Electronic Data Capture (EDC) clinical research support system. The Alliance Clinical Research Support System will be used as the EDC system, and random allocation factors for age (older than 65 years or younger than 65 years) and FAC (scores of 0–1 or ≧2) will be set at baseline. Regarding the blinding technique, only the primary outcome measure (FAC score) will be blinded using third-party assessment. Specifically, video-based FAC assessment will be performed by a third-party physical therapist who will not be involved in patient treatment or assessment and who will be blinded to the allocation. These measures will be implemented to reduce biases, improve the quality of the research, and ensure comparability. It will not be possible to use the blinding technique among the participants (patients) and the treatment providers (physical therapists) as they will inevitably know the allocation group. Therefore, it will be difficult to use the placebo technique in this study.

### Treatments

#### Gait Treatment Using HAL and CGT

The HAL group will undergo a total of nine gait treatment programs once a day for 20 min. The gait treatment program should be conducted at least once weekly, but we will recommend it twice or thrice per week. There is no provision for implementing a gait treatment program for two consecutive days; however, it will be implemented while checking for the presence and degree of muscle pain, arthralgia, and fatigue. In the HAL group, the gait treatment program consists of a walking activity for 20 min (including breaks) while wearing the lower-limb version of HAL for a single leg or both legs. The goal of the gait treatment is to improve independent walking, gait speed, endurance, gait stability, and gait symmetry. Walking speed and walking distance are expected to gradually increase according to the durability and fatigue level (corrected Borg scale, etc.) of each participant. The patients in the HAL group will always use a mobile suspension system (All-In-One Walking Trainer) to prevent from falling during the walking activity. Conversely, participants in the CGT group will use a mobile suspension system (All-In-One Walking Trainer) as needed. HAL treatment requires one physical therapist specializing in stroke rehabilitation and one technical staff member to operate the mobile suspension system (All-In-One Walking Trainer). The program of the CGT group consists of walking for 20 min (including breaks) with a physical therapist using lower-limb orthoses and walking aids as needed. In the control group, CGT encompasses the same activity without the HAL system but with the physical therapist being in charge of the patient. CGT requires one physical therapist specializing in stroke rehabilitation and another physical therapist or technical staff member to ensure safety when using a mobile suspension system (All-In-One Walking Trainer) or when the patient has severe gait disturbance. In both groups, the actual walking time and distance (excluding breaks) during the 20-min walking activity will be recorded.

#### HAL System and Treatment

**FIGURE 1:**
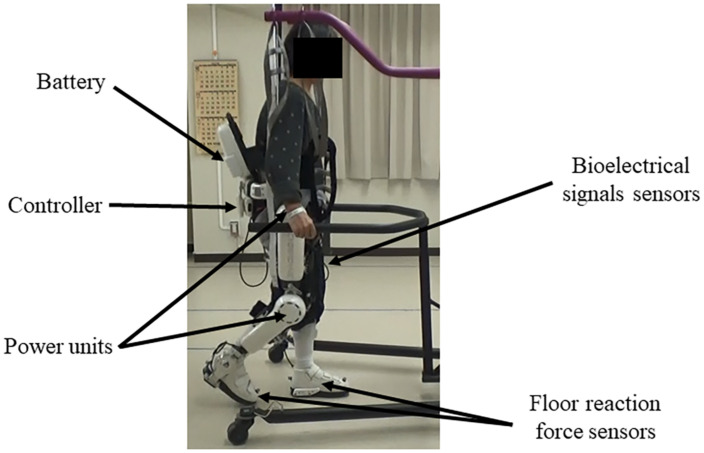
Example of gait treatment using the wearable cyborg HAL on the paretic lower limb. HAL is composed of an exoskeletal frame, power units, a battery, a controller, BES sensors, and floor reaction sensors, together with belts to secure the waist, thigh, and lower leg. HAL, Hybrid Assistive Limb; BES, bioelectrical signals.

**FIGURE 2:**
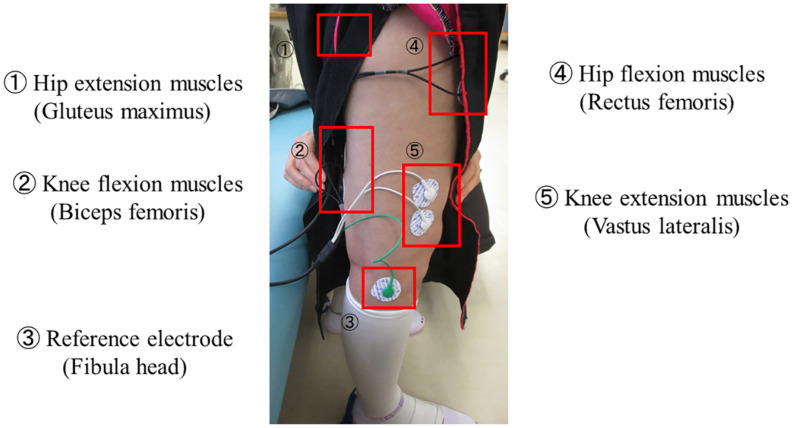
Example of BES sensor. Electrodes are attached to the surface of the wearer’s skin over the hip and knee flexion and extension muscles to detect the nerve and muscle action potentials as BES. BES, bioelectrical signals.

HAL is composed of an exoskeletal frame, power units, a battery, a controller, BES sensors, and floor reaction force sensors, together with belts to secure the waist, thigh, and lower leg (Figure 1; Kawamoto et al., 2009; Watanabe et al., 2020). Detailed information on the HAL system is available in previous reports (Kawamoto and Sankai, 2002; Lee and Sankai, 2005; Kawamoto et al., 2009; Watanabe et al., 2014, 2020; Sankai and Sakurai, 2018). The weight of the single-leg HAL version is approximately 9 kg, whereas that of the two-leg version is approximately 14 kg. Electrodes are attached to the surface of the wearer’s skin over the rectus femoris, gluteus maximus, vastus lateralis, and biceps femoris muscles to detect the nerve and muscle action potentials as BES (Figure 2). The single-leg HAL version will be attached to the paretic lower limb, and the Cybernic Voluntary control (CVC) mode will be selected (Kawamoto et al., 2009). In the CVC mode, HAL provides assistive joint torque in accordance with the detected BES of agonistic/antagonistic pair for each joint in real time. This can be summarized in the following equation: T = G_f × A_f - G_e × A_e, where T is the assist torque, G_f and G_e are the gain parameters of respectively the flexor and extensor muscles, and A_f and A_e are the detected amplitude of BES of each muscle after filtration of surface electric activity. This equation is applicable to each of the hip and knee joints. G_f and G_e are configured for each patient’s comfort during training in each session. However, if BES in the patients are not detectable due to severe hemiplegia, the Cybernic Autonomous Control mode will be selected (Kawamoto and Sankai, 2005).

During practical treatment, medical staff, especially physical therapists and doctors, who have received HAL safety training in advance are the only personnel who will have permission to operate the HAL system and apply it to the patients. For assistive settings in which the HAL system controller is used, the physical therapists will adjust the sensitivity level, torque tuner and balance tuner, the upper limit of the assist torque, and joint angle range. The sensitivity level allows the physical therapists to select the filter to process the BES and the amplification factor. The filter can be selected from among two levels (L1 = normal sensitivity; L2 = higher sensitivity). The upper limit of the assist torque can be changed by 10% in the range of 0–100%. The limit of joint angles can be configured by 5° in the range from 70° to 120° for hip flexion, −20° to 40° for hip extension, 70° to 120° for knee flexion, and −5° to 40° for knee extension. The joint angle range covers the joint angles required for standing up and walking. If the patient has a limited range of motion in the lower limbs, adjusting the range of motion of the HAL joints can reduce the risk of excessive assistance. These adjustments are made by the physical therapists. The adjustment of torque tuner and balance tuner is only for the CVC mode. In this mode, the torque tuner can be used to adjust the strength of the assist torque and the balance tuner can be used to adjust the flexion/extension assist torque ratio. In brief, the physical therapists will adjust the power and balance of the assist torque based on the given information, such as BES on the controller screen and gait posture during walking activity. Moreover, patients who need support for the lower extremities on the non-paralyzed side will be fitted with HAL for both legs and the appropriate type of HAL mode will be selected. Depending on the condition of the patient’s non-paretic lower limb, we will select HAL for both legs or a single leg. The rehabilitation doctor and physical therapist specializing in stroke will consider the appropriate type of HAL. These mechanisms enable the HAL system to coordinate an appropriate level and timing of torque to assist the hip and knee joint motions (Lee and Sankai, 2005; Sankai and Sakurai, 2018).

#### Permitted and Prohibited Concomitant Treatments

In terms of physical therapy, any RAGT other than HAL will be prohibited during the intervention period. Regarding physical therapy other than the intervention, the following physical therapies will be performed according to the patient’s condition: normal exercise therapy (e.g., range of motion exercise, muscle strengthening exercise, endurance strengthening exercise, and coordinated exercise), voluntary facilitation exercise, lower-limb stretching, basic movement exercise, sitting and standing balance practice, endurance practice (e.g., bicycle ergometer), conventional overground gait training, and stair climbing practice. In both groups, a physical therapist from our stroke care team will perform other training interventions, which will take approximately 40–60 min on weekdays, excluding holidays and public holidays. The time taken will be recorded and checked later for any differences between the two groups. The amount and content of the interventions for other occupational and speech therapies are not specified and will just be implemented as needed.

### Outcomes

#### Primary Outcome Measure

The FAC score will be assessed as the primary outcome measure (Holden et al., 1986). The FAC distinguishes 6 levels of walking ability on the basis of the amount of physical support required (Holden et al., 1984, 1986; Mehrholz et al., 2007). The 6 levels of walking ability of the patient are as follows.

• FAC score 0: “Non-functional Ambulator” – The patient classified in this level is in a serious condition such as those who are bedridden, therefore, they cannot sit, stand, and etc. A patient can only ambulate in parallel bars, or requires supervision or physical assistance from more than one person to ambulate safely outside of parallel bars.
• FAC score 1: “Ambulator-Dependent for Physical Assistance-Level II” – A patient requires manual contact of no more than one person during ambulation to prevent falling. Manual contact is continuous and necessary to support body weight as well as to maintain balance or assist coordination.
• FAC score 2: “Ambulator-Dependent for Physical Assistance-Level I” – A patient requires manual contact from no more than one person during ambulation to prevent falling. Manual contact consists of continuous or intermittent light touch to assist balance or coordination.
• FAC score 3: “Ambulator-Dependent for Supervision” – A patient can ambulate on level surfaces without manual contact of another person but, for safety, requires stand-by guarding of no more than one person because of poor judgment, questionable cardiac status, or the need for verbal cuing to complete the task.
• FAC score 4: “Ambulator-Independent, Level Surfaces Only” – A patient can ambulate independently on level surfaces, but requires supervision or physical assistance to any of the following: stairs, inclines, or non-level surfaces.
• FAC score 5: “Ambulator-Independent” – A patient can ambulate independently on non-level and level surfaces, stairs, and inclines.

Our previous pilot study suggested that FAC has a higher effect size than other outcome measures (Watanabe et al., 2020; Ueno et al., 2021). Therefore, the FAC score was selected as the primary endpoint. Furthermore, we will use third-party evaluation by recording a video to ensure the objectivity of the FAC evaluation; the third-party evaluator will be blinded. Two physical therapists who are not involved in patient treatment will evaluate the video independently. If the scores are different, one physical therapist will consult with another physical therapist and the two will decide on one score. The same physical therapist will perform the evaluation for the same patient. In this study, we will use objective evaluation results in a central review rather than actual on-site evaluations.

#### Secondary Outcome Measures

The following parameters will be assessed as secondary outcomes measures: Mini-Mental State Examination (0–30 points), Brunnstrom recovery stage of the lower limbs (1–6 stage), Fugl–Meyer assessment of the lower limbs (motor function domain, 0–34 points) (Fugl-Meyer et al., 1975), 6-min walking distance (m) (Kosak and Smith, 2005), comfortable gait speed (m/s), step length (m), cadence (steps/min), Barthel Index (0–100 points), Functional Independence Measure (0–126 points), gait posture, motion analysis (muscle activity), amount of activity (evaluated using an activity meter), stroke-specific QOL (SS-QOL), and modified Rankin Scale score (0–6 scale).

#### Safety Outcome Measurement

The safety of this study will be assessed based on vital signs, physical examinations, and disease occurrence status. There is a risk of injury because of falls; therefore, a fall-prevention device (All-In-One Walking Trainer) will be used if necessary. The disease occurrence status in this study is defined as worsening of undesirable or unintended diseases, disorders, signs, symptoms, or medical conditions occurring in the study participants. If a disease occurrence status is detected, the investigator will immediately take appropriate measures and file a consistent medical record and case report. If the disease occurrence status is attributed to a mechanical problem with the HAL system, the investigator will check the records for errors in the history of the device’s operation and confirm the test equipment failure.

### Participant Timeline (Figure 3)

This study will include three phases: (1) pre-observation phase (patient enrollment, baseline assessment, and randomization); (2) treatment phase [the patients will undergo a total of nine sessions, twice or thrice per week over 3−4 weeks; the HAL and control groups will perform gait treatment with HAL and CGT, i.e., walking aids and gait orthoses, respectively, as follows: initial treatment phase evaluation, 1st interim evaluation (before the 4th intervention), and 2nd interim evaluation (before the 7th intervention)]; and, finally (3) post-treatment evaluation phase (after the 9th intervention). Subsequently, a 6-month post-treatment evaluation (follow-up assessment) will be conducted to assess body functions, walking ability, ADL, and QOL in the post-observation phase.

**FIGURE 3:**
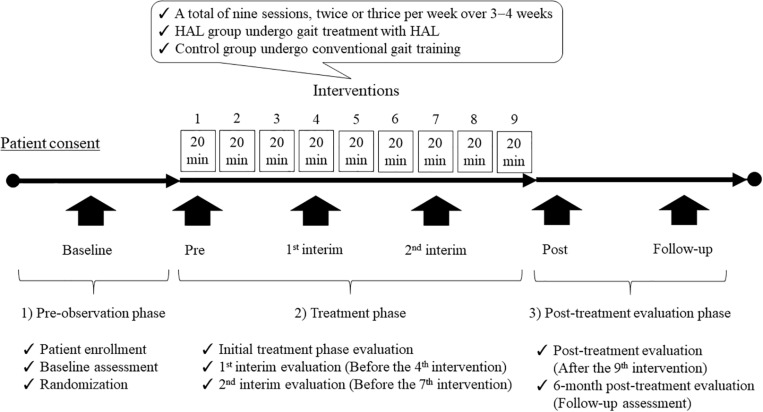
Participant timeline of the study. This single-center, randomized, parallel-group, controlled trial will include three phases: (1) pre-observation phase (patient enrollment, baseline assessment, and randomization); (2) treatment phase [patients will undergo a total of nine sessions, twice or thrice per week over 3–4 weeks; the HAL and control groups will undergo gait treatment with HAL and CGT, i.e., walking aids and gait orthoses, respectively, as follows: initial treatment phase evaluation, 1st interim evaluation (before the 4th intervention), and 2nd interim evaluation (before the 7th intervention)]; and, finally (3) post-treatment evaluation phase (after the 9th intervention). Subsequently, a 6-month post-treatment evaluation (follow-up assessment) will be conducted to evaluate body functions, walking ability, ADL, and QOL in the post-observation phase. HAL, Hybrid Assistive Limb; CGT, conventional gait training; ADL, activities of daily living; QOL, quality of life.

### Criteria for Discontinuation (Discontinuation of Protocol Treatment)

1) When a patient requests to decline participation in the study or withdraws consent.
2) If it turns out that a patient is not satisfied with his or her eligibility after registration.
3) When it is judged that it is not preferable to continue using the test equipment due to worsening of the underlying disease.
4) When it is difficult to continue the study due to exacerbation of complications.
5) When it is difficult to continue the study due to illness or other reasons.
6) When the entire study is discontinued.
7) When the doctor deems it inappropriate to continue the study for other reasons.

### Data Management

The research data will be saved in a personal computer of the Center for Innovated Medicine and Engineering at the University of Tsukuba Hospital, and a back-up copy will be stored in an external hard disk. Computer access will require a password to prevent third-party viewing. The researcher will be responsible for data storage endeavors aimed at ensuring that the research documents will not be lost, discarded, or stolen over 10 years.

### Data Monitoring

The investigator will appoint a person who is not directly involved in the clinical trial to act as the monitor, who will be responsible for regularly monitoring data quality, written informed consent documents, protocol compliance, and overall clinical trial progress during the study, as well as reporting these items to the investigator in writing. The monitoring work will be conducted by the staff of the University of Tsukuba, Tsukuba Clinical Research & Development Organization.

### Sample Size

We calculated the sample size of this study based on our exploratory study (Watanabe et al., 2020), the results of which led us to estimate that the mean difference of the change in the intervention group at the end of the FAC was 0.82 and the combined SD was 0.63. However, the 50% lower confidence limit of the mean difference, 0.63, was used conservatively in consideration of the small sample size and design difference of the exploratory study. The minimum sample size necessary for the power of the primary analysis to exceed 0.8 was estimated as 34 in two groups. Considering information loss because of missingness, the target number of cases was set to 40 in two groups.

### Statistical Analysis

The target population in this analysis will be all participants who have undergone at least one intervention during the study period, including the pre-observation period. The significance level will be 5% on both sides, and the confidence level will be 95%. We will test differences in the baseline variables between the HAL and CGT groups using the Fisher exact test for categorical data and Mann–Whitney *U* test for continuous data. The primary analysis will consist of mixed effect models for repeated measures (Mallinckrodt et al., 2001) to reduce bias due to dropouts, in which changes in FAC at the 4th, 7th, and 9th interventions and at 6 months from the baseline will be included as the outcome variable. Whereas, group, time point, and the group × time point interaction will be included as the fixed effects. Using this model, a *t*-test will be performed on the difference in the adjusted mean between the intervention groups at the time of the end-of-term evaluation. In this analysis, we do not plan to conduct a preliminary test for the diagnosis of normality because of robustness of the statistical model with the normal distribution (Heeren and D’Agostino, 1987; Maruo et al., 2017) and the inflation of type I error due to the preliminary tests (Shuster, 2005, 2009). For other analyses, summary statistics will be calculated for the patient background factors. The summary statistics refer to the number of cases, mean value, standard deviation, minimum value, median value, and maximum value for continuous variables and the number of cases and their ratios for categorical variables. For the primary and secondary endpoints, the summary statistics will be calculated for each evaluation time point. For the primary analysis, we will estimate the differences between the groups at different time points. An analysis similar to the primary analysis will be performed for the secondary continuous outcome measures. All statistical analyses will be conducted using IBM SPSS version 26.0 (IBM Corp., Armonk, NY, United States) and SAS version 9.4 (SAS Institute Inc., Cary, NC, United States).

## Discussion

This study will be an RCT aiming to clarify the efficacy and safety of gait treatment using HAL versus CGT for hemiplegic patients with acute stroke. In addition, we will establish a protocol for doctor-initiated clinical trials of an innovative medical device for acute stroke based on the results of this study. If the results reveal that HAL has greater effectiveness than CGT in improving independent walking in patients with hemiplegic acute stroke, this study will promote the treatment of these patients using the HAL system as an effective tool in future stroke rehabilitation programs.

The following medical and social effects will be recognized by performing HAL treatment among patients with acute stroke hemiplegia and improving independent walking: (1) improvement of the function of the lower limbs on the paralyzed side and walking ability; (2) improvement of ADL and QOL can be expected by improving independent walking; (3) promotion of social participation; (4) reduction of the burden on the caregiver; (5) shortening of the length of hospital stay and the time required to achieve independent walking, which may lead to a reduction of medical expenses.

The conventional RAGT is problematic as it tends to provide passive assistance (Lotze, 2003; Kaelin-Lang et al., 2005; Israel et al., 2006; Koenig et al., 2011). The wearable cyborg lower-limb version of HAL used in this study assists the person according to the exercise intention. The hip and knee joint motors are driven to provide active assistance (Kawamoto et al., 2009). The HAL system, which supports appropriate muscle activity, walking, and physical exercise based on BES acquired from voluntary movements, is an innovative robotic technology not present in other treatment methods. In addition, exercise and gait treatment, which cannot be performed by standard treatment, are possible, and motor function and walking ability may be improved. The amount and intensity of gait training are important factors in gait reconstruction among patients with stroke, and factors such as repeated movements, difficulty in adjustments, rewards, and feedback are important for motor learning (Lotze, 2003; Kwakkel et al., 2004; Krakauer, 2006; Huang and Krakauer, 2009; Krakauer and Mazzoni, 2011; Shishov et al., 2017). An interactive biofeedback is generated when using the HAL system, and it is possible that the motor learning effect is high (Morishita and Inoue, 2016; Sankai and Sakurai, 2018). Therefore, HAL treatment may be an innovative gait treatment that improves independent walking.

In the present study, FAC was selected as the primary outcome because our previous pilot study suggested that FAC showed a higher effect size than other outcome measures (Watanabe et al., 2020; Ueno et al., 2021). FAC is the most common tool used as a primary outcome to assess the effect of RAGT (Geroin et al., 2013). A recent systematic review of electromechanical-assisted gait training in combination with physical therapy showed that it increased the odds of participants achieving independent walking [odds ratio (random effects), 2.01; 95% confidence interval, 1.51–2.69; 38 studies, 1567 participants; *P* < 0.00001; I^2^ = 0%; high-quality evidence] (Mehrholz et al., 2020). In this study, two physical therapists who are not involved in patient assessment and treatment will independently perform the video evaluation. Moreover, we will use objective evaluation results in a central review rather than actual on-site evaluations. It will be possible to objectively evaluate the independent walking of patients by adopting video evaluation for FAC evaluation.

A previous RCT reported no differences between the HAL and control groups for any outcome, including the FAC score after the intervention or at 6 months after stroke, despite the extra resources required for the HAL training (Wall et al., 2020). Among the patients with an FAC score of 0–1 who participated in the previous RCT, approximately 70% had an FAC of 0 and severe gait disturbance. The average time from the onset of stroke was approximately 5 weeks, which corresponded to the subacute stage (within 5 weeks after stroke onset), and the patients were relatively young (16–67 years). The intervention was conducted 4 times per week, 16 times in total for 4 weeks, and HAL treatment was performed on a treadmill (Wall et al., 2020). In contrast, the present RCT will target patients with an FAC score of 0–2 within 3 weeks of stroke onset. The inclusion criteria for this study include patients who can undergo gait training using a mobile suspension system (All-In-One Walking Trainer) and patients who can walk even with an FAC score of 0. In addition, age and FAC scores will be used as allocation factors to equalize the age and walking ability between the two groups. At present, it is not possible to discuss whether a treadmill or overground gait training is a good intervention method for HAL treatment, but we have selected overground gait training, which is similar to normal walking. In addition, a recent Cochrane report described that the evidence showing the effectiveness of treadmill training for walking after stroke was low-to-moderate, even after many years of research in the field (Mehrholz et al., 2017). Thus, we consider that a combination of treadmill and HAL may not fully utilize the potential of HAL training. Because overground gait training includes a change of direction, we believe that overground gait training with HAL can be performed in a form that is closer to real-life walking.

The present study has several limitations. First, this study is an RCT comparing groups using the minimization method; however, because of the small sample size, parameters other than the allocation factors (FAC and age at baseline) may be imbalanced between the two groups. Second, this study is not a multicenter trial but a single–institution trial, and the results are unlikely to be generalized. Third, it is not possible to blind the participants and the treatment providers (physical therapists); therefore, in this study, it is not possible to perform sham interventions or set up a third-party physical therapist.

## Data Availability Statement

The original contributions presented in the study are included in the article/supplementary material, further inquiries can be directed to the corresponding author.

## Ethics Statement

The studies involving human participants were reviewed and approved by the Tsukuba University Clinical Research Review Board on October 6, 2020 (TCRB20-011). The study protocol was registered with the Japan Registry of Clinical Trials on October 14, 2020 (jRCTs032200151). The participants provided their written informed consent to participate in this study.

## Author Contributions

HW and AM: conceptualization, funding acquisition, and writing of the original draft. HW, AM, HK, YS, SK, TH, MS, YI, MH, HT, and KM: methodology and project administration. AM: review and editing. AM, HK, YS, SK, TH, MS, YI, MH, HT, KM, YH, EI, and YM: confirming the final manuscript. All authors contributed to the article and approved the submitted version.

## Conflict of Interest

The authors declare that the research was conducted in the absence of any commercial or financial relationships that could be construed as a potential conflict of interest.

## References

[B1] Campbell B. C. V. Khatri P. (2020). Stroke. *Lancet* 396 129–142. 10.1016/S0140-6736(20)31179-X 32653056

[B2] Dobkin B. H. (2005). Clinical practice. Rehabilitation after stroke. *N. Engl. J. Med.* 352 1677–1684. 10.1056/NEJMcp043511 15843670 PMC4106469

[B3] Duncan P. W. Sullivan K. J. Behrman A. L. Azen S. P. Wu S. S. Nadeau S. E. (2011). Body-weight-supported treadmill rehabilitation after stroke. *N. Engl. J. Med.* 364 2026–2036. 10.1056/NEJMoa1010790 21612471 PMC3175688

[B4] Fryer C. E. Luker J. A. McDonnell M. N. Hillier S. L. (2016). Self management programmes for quality of life in people with stroke. *Cochrane Database Syst. Rev.* 8:CD010442. 10.1002/14651858 27545611 PMC6450423

[B5] Fugl-Meyer A. R. Jääskö L. Leyman I. Olsson S. Steglind S. (1975). The post-stroke hemiplegic patient. 1. a method for evaluation of physical performance. *Scand. J. Rehabil. Med.* 7 13–31. 1135616

[B6] Fulk G. D. He Y. Boyne P. Dunning K. (2017). Predicting home and community walking activity poststroke. *Stroke* 48 406–411. 10.1161/STROKEAHA.116.015309 28057807

[B7] GBD 2016 Lifetime Risk of Stroke Collaborators (2018). Global, regional, and country-specific lifetime risks of stroke, 1990 and 2016. *N. Engl. J. Med.* 379 2429–2437. 10.1056/NEJMoa1804492 30575491 PMC6247346

[B8] Geroin C. Mazzoleni S. Smania N. Grandlfi M. Bonaiuti D. Gasperini G. (2013). Systematic review of outcome measures of walking training using electromechanical and robotic devices in patients with stroke. *J. Rehabil. Med.* 45 987–996. 10.2340/16501977-1234 24150661

[B9] Heeren T. D’Agostino R. (1987). Robustness of the two independent samples t−test when applied to ordinal scaled data. *Stat. Med.* 6 79–90. 10.1002/sim.4780060110 3576020

[B10] Hesse S. Schmidt H. Werner C. Bardeleben A. (2003). Upper and lower extremity robotic devices for rehabilitation and for studying motor control. *Curr. Opin. Neurol.* 16 705–710. 10.1097/01.wco.0000102630.16692.38 14624080

[B11] Holden M. K. Gill K. M. Magliozzi M. R. (1986). Gait assessment for neurologically impaired patients. Standards for outcome assessment. *Phys. Ther.* 66 1530–1539. 10.1093/ptj/66.10.1530 3763704

[B12] Holden M. K. Gill K. M. Magliozzi M. R. Nathan J. Piehl-Baker L. (1984). Clinical gait assessment in the neurologically impaired. Reliability and meaningfulness. *Phys. Ther.* 64 35–40. 10.1093/ptj/64.1.35 6691052

[B13] Huang V. S. Krakauer J. W. (2009). Robotic neurorehabilitation: a computational motor learning perspective. *J. Neuroeng. Rehabil.* 6:5. 10.1186/1743-0003-6-5 19243614 PMC2653497

[B14] Israel J. F. Campbell D. D. Kahn J. H. Hornby T. G. (2006). Metabolic costs and muscle activity patterns during robotic- and therapist-assisted treadmill walking in individuals with incomplete spinal cord injury. *Phys Ther.* 86 1466–1478. 10.2522/ptj.20050266 17079746

[B15] Jørgensen H. S. Nakayama H. Raaschou H. O. Olsen T. S. (1995a). Recovery of walking function in stroke patients: the Copenhagen Stroke Study. *Arch. Phys. Med. Rehabil.* 76 27–32. 10.1016/s0003-9993(95)80038-7 7811170

[B16] Jørgensen H. S. Nakayama H. Raaschou H. O. Vive-Larsen J. Støier M. Olsen T. S. (1995b). Outcome and time course of tecovery in stroke. part II: time course of recovery. The Copenhagen Stroke Study. *Arch. Phys. Med. Rehabil.* 76 406–412. 10.1016/s0003-9993(95)80568-0 7741609

[B17] Kaelin-Lang A. Sawaki L. Cohen L. G. (2005). Role of voluntary drive in encoding an elementary motor memory. *J. Neurophysiol.* 93 1099–1103. 10.1152/jn.00143.2004 15456807

[B18] Kawamoto H. Sankai Y. (2002). “Power assist system HAL-3 for gait disorder person,” in *Proceedings of the 8th International Conference on Computers Helping People with Special Needs (ICCHP 2002)*, (Berlin: Springer-Verlag), 196–203. 10.1007/3-540-45491-8_43

[B19] Kawamoto H. Sankai Y. (2005). Power assist method based on phase sequence and muscle force condition for HAL. *Adv. Robot.* 19 717–734. 10.1163/1568553054455103

[B20] Kawamoto H. Hayashi T. Sakurai T. Eguchi K. Sankai Y. (2009). Development of single leg version of HAL for hemiplegia. *Annu. Int. Conf. IEEE Eng. Med. Biol. Soc.* 2009 5038–5043. 10.1109/IEMBS.2009.5333698 19964376

[B21] Koenig A. Omlin X. Bergmann J. Zimmerli L. Bolliger M. Müller F. (2011). Controlling patient participation during robot-assisted gait training. *J. Neuroeng. Rehabil.* 8:14. 10.1186/1743-0003-8-14 21429200 PMC3076234

[B22] Kosak M. Smith T. (2005). Comparison of the 2-, 6-, and 12-minute walk tests in patients with stroke. *J. Rehabil. Res. Dev.* 42 103–107. 10.1682/jrrd.2003.11.0171 15742254

[B23] Krakauer J. W. (2006). Motor learning: its relevance to stroke recovery and neurorehabilitation. *Curr. Opin. Neurol.* 19 84–90. 10.1097/01.wco.0000200544.29915.cc 16415682

[B24] Krakauer J. W. Mazzoni P. (2011). Human sensorimotor learning: adaptation, skill, and beyond. *Curr. Opin. Neurobiol.* 21 636–644. 10.1016/j.conb.2011.06.012 21764294

[B25] Kwakkel G. Peppen R. V. Wagenaar R. C. Dauphinee S. W. Richards C. Ashburn A. (2004). Effects of augmented exercise therapy time after stroke: a meta-analysis. *Stroke* 35 2529–2539. 10.1161/01.STR.0000143153.76460.7d 15472114

[B26] Kwakkel G. Wagenaar R. C. Twisk J. W. Lankhorst G. J. Koetsier J. C. (1999). Intensity of leg and arm training after primary middle-cerebral-artery stroke: a randomised trial. *Lancet* 354 191–196. 10.1016/S0140-6736(98)09477-X 10421300

[B27] Langhorne B. J. Kwakkel G. (2011). Stroke rehabilitation. *Lancet* 377 1693–1702. 10.1016/S0140-6736(11)60325-5 21571152

[B28] Langhorne P. Coupar F. Pollock A. (2009). Motor recovery after stroke: a systematic review. *Lancet Neurol.* 8 741–754. 10.1016/S1474-4422(09)70150-4 19608100

[B29] Lee S. Sankai Y. (2005). Virtual impedance adjustment in unconstrained motion for an exoskeletal robot assisting the lower limb. *Adv. Robot.* 19 773–795. 10.1163/1568553054455095

[B30] Lotze M. (2003). Motor learning elicited by voluntary drive. *Brain* 126 866–872. 10.1093/brain/awg079 12615644

[B31] Mallinckrodt C. H. Clark W. S. David S. R. (2001). Accounting for dropout bias using mixed-effects models. *J. Biopharm. Stat.* 11 9–21. 10.1081/BIP-100104194 11459446

[B32] Maruo K. Yamaguchi Y. Noma H. Gosho M. (2017). Interpretable inference on the mixed effect model with the Box-Cox transformation. *Stat. Med.* 36 2420–2434. 10.1002/sim.7279 28294388

[B33] Mehrholz J. Pohl M. Platz T. Kugler J. Elsner B. (2018). Electromechanical and robot-assisted arm training for improving activities of daily living, arm function, and arm muscle strength after stroke. *Cochrane Database Syst. Rev.* 9:CD006876. 10.1002/14651858.CD006876.pub5 PMC6513114 30175845

[B34] Mehrholz J. Thomas S. Elsner B. (2017). Treadmill training and body weight support for walking after stroke. *Cochrane Database Syst. Rev.* 8:CD002840. 10.1002/14651858.CD002840.pub4 PMC6483714 28815562

[B35] Mehrholz J. Thomas S. Kugler J. Pohl M. Elsner B. (2020). Electromechanical-assisted training for walking after stroke. *Cochrane Database Syst. Rev.* 10:CD006185. 10.1002/14651858.CD006185.pub5 PMC8189995 33091160

[B36] Mehrholz J. Wagner K. Rutte K. Meissner D. Pohl M. (2007). Predictive validity and responsiveness of the functional ambulation category in hemiparetic patients after stroke. *Arch. Phys. Med. Rehabil.* 66 1530–1539. 10.1016/j.apmr.2007.06.764 17908575

[B37] Ministry of Health Labour and Welfare (2019a). *Results of vital statistics, 2019.* Available online at: https://www.mhlw.go.jp/toukei/saikin/hw/jinkou/geppo/nengai19/dl/gaikyouR1.pdf (accessed January 16, 2021).

[B38] Ministry of Health Labour and Welfare (2019b). *The results of Comprehensive Survey of Living Conditions, 2019.* Available online at: https://www.mhlw.go.jp/toukei/saikin/hw/k-tyosa/k-tyosa19/dl/05.pdf (accessed January 16, 2021).

[B39] Morishita T. Inoue T. (2016). Interactive bio-feedback therapy using hybrid assistive limbs for motor recovery after stroke: current practice and future perspectives. *Neurol. Med. Chir.* 56 605–612. 10.2176/nmc.st.2016-0094 27616320 PMC5066081

[B40] Rigby H. Gubitz G. Phillips S. (2009). A systematic review of caregiver burden following stroke. *Int. J. Stroke.* 4 285–292. 10.1111/j.1747-4949.2009.00289.x 19689757

[B41] Sankai Y. Sakurai T. (2018). Exoskeletal cyborg-type robot. *Sci. Robot.* 3:eaat3912. 10.1126/scirobotics.aat3912 33141743

[B42] Sczesny-Kaiser M. Trost R. Aach M. Schildhauer T. A. Schwenkreis P. Tegenthoff M. (2019). A randomized and controlled crossover study investigating the improvement of walking and posture functions in chronic stroke patients using HAL exoskeleton - The HALESTRO Study (HAL-Exoskeleton STROke Study). *Front. Neurosci.* 13:259. 10.3389/fnins.2019.00259 30983953 PMC6450263

[B43] Shishov N. Melzer I. Bar-Haim S. (2017). Parameters and measures in assessment of motor learning in neurorehabilitation; A systematic review of the literature. *Front. Hum. Neurosci.* 11:82. 10.3389/fnhum.2017.00082 28286474 PMC5324661

[B44] Shuster J. J. (2005). Diagnostics for assumptions in moderate to large simple clinical trials: Do they really help? *Stat. Med.* 24 2431–2438. 10.1002/sim.2175 15977289

[B45] Shuster J. J. (2009). Student t -tests for potentially abnormal data. *Stat. Med.* 28 2170–2184. 10.1002/sim.3581 19326398 PMC3666168

[B46] Suzuki K. Mito G. Kawamoto H. Hasegawa Y. Sankai Y. (2007). Intention-based walking support for paraplegia patients with robot suit HAL. *Adv. Robot.* 21 1441–1469. 10.1163/156855307781746061

[B47] Taki S. Imura T. Iwamoto Y. Imada N. Tanaka R. Araki H. (2020). Effects of exoskeletal lower limb robot training on the activities of daily living in stroke patients: Retrospective pre-post comparison using propensity score matched analysis. *J. Stroke Cerebrovasc. Dis.* 29:105176. 10.1016/j.jstrokecerebrovasdis.2020.105176 32912532

[B48] Tsurushima H. Mizukami M. Yoshikawa K. Ueno T. Hada Y. Gosho M. (2019). Effectiveness of a walking program involving the hybrid assistive limb robotic exoskeleton suit for improving walking ability in stroke patients: Protocol for a randomized controlled trial. *JMIR Res. Protoc.* 8:e14001. 10.2196/14001 31605515 PMC6913690

[B49] Ueno T. Marushima A. Kawamoto H. Shimizu Y. Watanabe H. Kadone H. (2021). Staged treatment protocol for gait with hybrid assistive limb in the acute phase of patients with stroke. *Assist. Technol.* 2021:1862361. 10.1080/10400435.2020.1862361 33465002

[B50] Waddell K. J. Strube M. J. Bailey R. R. Klaesner J. W. Birkenmeier R. L. Dromerick A. W. (2017). Does task-specific training improve upper limb performance in daily life poststroke? *Neurorehabil. Neural. Repair.* 31 290–300. 10.1177/1545968316680493 27909071 PMC5481638

[B51] Wall A. Borg J. Vreede K. Palmcrantz S. (2020). A randomized controlled study incorporating an electromechanical gait machine, the Hybrid Assistive Limb, in gait training of patients with severe limitations in walking in the subacute phase after stroke. *PLoS One* 15:e0229707. 10.1371/journal.pone.0229707 32109255 PMC7048283

[B52] Watanabe H. Goto R. Tanaka N. Matsumura A. Yanagi H. (2017). Effects of gait training using the Hybrid Assistive Limb^®^ in recovery-phase stroke patients: a 2-month follow-up, randomized, controlled study. *NeuroRehabilitation* 40 363–367. 10.3233/NRE-161424 28222558

[B53] Watanabe H. Marushima A. Kadone H. Ueno T. Shimizu Y. Kubota S. (2020). Effects of gait treatment with a single-leg hybrid assistive limb system after acute stroke: A non-randomized clinical trial. *Front. Neurosci.* 13:1389. 10.3389/fnins.2019.01389 32038125 PMC6987474

[B54] Watanabe H. Tanaka N. Inuta T. Saitou H. Yanagi H. (2014). Locomotion improvement using a hybrid assistive limb in recovery phase stroke patients: a randomized controlled pilot study. *Arch. Phys. Med. Rehabil.* 95 2006–2012. 10.1016/j.apmr.2014.07.002 25010538

[B55] Yokota C. Yamamoto Y. Kamada M. Nakai M. Nishimura K. Ando D. (2019). Acute stroke rehabilitation for gait training with cyborg type robot Hybrid Assistive Limb: A pilot study. *J. Neurol. Sci.* 404 11–15. 10.1016/j.jns.2019.07.012 31323516

